# Serum Retinol and Carotenoids in Association with Biomarkers of Insulin Resistance among Premenopausal Women

**DOI:** 10.5402/2013/619516

**Published:** 2012-09-12

**Authors:** Stacy A. Blondin, Edwina H. Yeung, Sunni L. Mumford, Cuilin Zhang, Richard W. Browne, Jean Wactawski-Wende, Enrique F. Schisterman

**Affiliations:** ^1^Epidemiology Branch, Division of Epidemiology, Statistics, and Prevention Research, Eunice Kennedy Shriver National Institute of Child Health and Human Development, Bethesda, MD 20892, USA; ^2^Center for Human Nutrition, Department of International Health, Johns Hopkins Bloomberg School of Public Health, Baltimore, MD 21205, USA; ^3^Department of Biotechnical and Clinical Laboratory Sciences, University at Buffalo, Buffalo, NY 14214, USA; ^4^Department of Social and Preventive Medicine, University at Buffalo, Buffalo, NY 14214, USA

## Abstract

*Objective*. The aim of this study was to investigate how serum retinol and carotenoids (**β**-carotene, **β**-cryptoxanthin, lutein/zeaxanthin, lycopene) are associated with biomarkers of insulin resistance. *Research Methods and Procedures*. The BioCycle Study (2005–2007) is a prospective cohort of 259 healthy premenopausal women. Fasting serum samples were collected at up to sixteen clinic visits, from which retinol, carotenoids, insulin, glucose, and sex hormone-binding globulin (SHBG) were measured. Insulin resistance was estimated by the homeostasis model assessment (HOMA-IR). Linear mixed models were used to determine associations adjusting for age, race, body mass index (BMI), education, smoking, physical activity, triglycerides, and energy intake. *Results*. Retinol was positively associated with HOMA-IR (*β* = 0.19 (95% CI: 0.07, 0.32)) units per ug/mL increase in retinol; the relationship was driven by insulin (*β* = 0.20 (95% CI: 0.08, 0.31)). Retinol was inversely associated with SHBG (*β* = −0.22 (95% CI: −0.28, −0.16)). Although no significant associations were found between serum carotenoids and HOMA-IR, **β**-carotene was positively associated with SHBG and **β**-cryptoxanthin inversely with fasting plasma glucose. *Conclusion*. Results indicate a possible role for serum retinol in the pathogenesis of type 2 diabetes. However, they do not support a strong association between individual or total serum carotenoids and insulin resistance.

## 1. Introduction

Vitamin A is an essential nutrient involved in myriad physiological functions, including gene expression, immunity, reproduction, and growth [1]. However, its role in glucose metabolism is less well understood. Vitamin A is obtained in the diet in two forms: preformed vitamin A as retinyl esters in animal foods and provitamin A as carotenoids (fat-soluble yellow, orange, and red pigments) in plant foods. Both forms serve as precursors to retinol, the alcoholic and predominant circulating form of vitamin A, which is transported in serum in a 1 : 1: 1 ratio with retinol binding protein (RBP) and transthyretin (TTR) [1]. 

Of the hundreds of plant-derived carotenoids, only *α*-carotene, *β*-carotene, *β*-cryptoxanthin, lutein, zeaxanthin, and lycopene are universally found in human sera and only provitamin A carotenoids (*α*-carotene, *β*-carotene, and *β*-cryptoxanthin) are fractionally converted to retinol [2]. Although serum retinol levels are under tight homeostatic control, serum carotenoid concentrations are responsive to dietary intake. However, neither is strictly determined by dietary intake, as their metabolism is influenced by age, gender, hormones, and genetics [3].

Despite the hypothesis that serum retinol and carotenoids may mitigate chronic disease pathogenesis via their anti-oxidative properties [4, 5], observational studies have produced inconsistent findings [6–15], and randomized controlled trials have shown no effect of varying doses of *β*-carotene supplements on risk of type 2 diabetes [16–19]. Moreover, positive associations between retinol binding protein (RBP) and metabolic disturbances [20–23] support the hypothesis that serum retinol may in fact be associated with impaired glucose metabolism [24]. Given the conflicting findings from previous studies, the associations for retinol and carotenoids with insulin resistance remain inconclusive.

Therefore, the present study investigated associations between food-sourced serum retinol and carotenoids and biomarkers of insulin resistance. To our knowledge, no study has considered these associations among premenopausal women not taking vitamin supplements. 

## 2. Materials and Methods

### 2.1. Study Design and Participants

The BioCycle Study is a prospective cohort study conducted between 2005 and 2007, designed to investigate the association between measures of oxidative stress and endogenous reproductive hormone levels [25, 26]. Healthy premenopausal women 18–44 years of age with self-reported menstrual cycle length between 21 and 35 days for the past 6 months were recruited. Exclusion criteria included history of chronic disease or gynecological abnormalities, current pregnancy or lactation, use of specified medications or antibiotics, and regular vitamin supplementation during the study period. A total of 259 women participated in the study; 250 were followed for 2 menstrual cycles and 9 for a single cycle. 

The University at Buffalo Health Sciences Institutional Review Board (IRB) approved the study and served as the IRB designated by the National Institutes of Health for this study under a reliance agreement. Written informed consent was obtained from all subjects.

### 2.2. Data Collection 

#### 2.2.1. Biomarkers

Morning fasting blood specimens were collected on up to eight occasions during the course of each menstrual cycle (according to menstrual cycle phases, timing assisted by use of fertility monitors) [25, 27]. Specimens were collected and aliquoted according to standardized protocols. Aliquots were frozen until the end of the study after which individual serum samples were analyzed in the same runs from each participant and then analyzed together to reduce batch variability. Serum insulin and SHBG were measured using a solid-phase competitive Chemiluminescent Enzymatic Immunoassay by Specialty Laboratories on the DPC Immulite 2000 analyzer (Siemens Medical Solutions Diagnostics). Interassay coefficients of variation were <8% for insulin and <10% for SHBG. Plasma glucose was assayed using a hexokinase-based methodology on a Beckman LX20 autoanalyzer (CV < 3%). Insulin resistance was calculated using the homeostasis model assessment (HOMA-IR) as: [fasting insulin (mU/L) ∗ fasting glucose (mmol/L)]/22.5 [28]. 

Serum retinol and serum carotenoids (*β*-carotene, *β*-cryptoxanthin, lutein/zeaxanthin, and lycopene) were measured simultaneously using HPLC [29]. The coefficients of variation were 6.1%, 7.5%, 8.0%, 5.6%, and 5.6%, respectively [29]. Total carotenoids were calculated as the sum of measured *β*-carotene, *β*-cryptoxanthin, lutein/zeaxanthin, and lycopene. Serum triglycerides, total cholesterol, and HDL cholesterol were determined by an auto chemistry analyzer (<5% CV for all assays), and LDL cholesterol was calculated using the Friedewald formula [30]. 

#### 2.2.2. Demographics

Participants provided information on age (using date of birth), race, smoking, education, and physical activity via questionnaire [25]. Smoking exposure status (current smoker; nonsmoker, passively exposed; nonsmoker, nonpassively exposed) combined information regarding individual smoking habits and second-hand exposure. Physical activity was assessed using the International Physical Activity Questionnaire (IPAQ); women were divided into low, moderate, and high categories according to standard IPAQ cutoffs [31]. At baseline, hip circumference, waist circumference, height, and weight were measured by trained staff using standardized protocols and used to calculate BMI and waist-to-hip ratio (WHR). 

#### 2.2.3. Dietary Assessment

Dietary intake was assessed via 24-hour dietary recall up to 4 times per menstrual cycle, for a total of up to 8 recalls per participant [32]. The timing of recall administration corresponded with menses, the mid-follicular phase, ovulation, and the midluteal phase, unique to each participant [32]. The Nutrition Data System for Research software version 2005 developed by the Nutrition Coordinating Center, University of Minnesota, Minneapolis, MN, was used to analyze dietary intake data [32]. The values for retinol, individual carotenoids, alcohol, fiber, fat, and total energy from subjects' completed 24-hour dietary recalls were averaged over both cycles for this analysis. Only one participant indicated noncompliance with abstaining from vitamin consumption during the study followup. However, all observations were kept in the analysis, as removing outlying values of carotenoid and retinol levels did not qualitatively alter findings. 

### 2.3. Statistical Analysis

Variables with nonnormal distributions were log-transformed and *P* values <0.05 were considered statistically significant. A minimum of 5 measurements were obtained per cycle for each woman, and less than 6% of more than 3900 repeated observations were missing for measurement of retinol or carotenoids. No differences were seen in demographic characteristics between those with and without missing data. 

Descriptive statistics were calculated for sociodemographic, lifestyle, and clinical characteristics according to tertile of serum retinol at baseline (menses visit during the first cycle). Chi-square (*χ* ^2^) and Fisher's exact tests were used to assess differences among groups across categorical variables, and analysis of variance (ANOVA) was used to test differences for continuous variables. Spearman correlation coefficients were used to assess relationships between baseline serum retinol and carotenoid levels, adjusted for age, race, and triglycerides.

Age- and multivariate-adjusted linear mixed models, accounting for up to 16 repeated measures per subject, were implemented to evaluate associations between serum retinol and carotenoids and HOMA-IR, insulin, glucose, and SHBG. Final models adjusted for age, race, BMI, education, smoking, physical activity, total energy intake, and triglycerides. Additional models were run which further adjusted for total cholesterol, alcohol, dietary fiber, and fat intake. Finally, alternative models were run with WHR substituted for BMI.

All analyses were performed with Statistical Analysis Systems statistical software package version 9.1 (SAS Institute, Cary, NC, USA).

## 3. Results

In general, women were relatively young (mean age: 27.3 years), of normal weight (mean BMI: 24.1 kg/m^2^), physically active (55% highly active), nonsmoking (96%), and predominantly white (60%). Median (interquartile range (IQR)) serum retinol and *β*-carotene levels were 0.38 (0.34–0.44) *μ*g/mL and 0.15 (0.10–0.23) *μ*g/mL, respectively. Increasing serum retinol tertiles were further associated with increasing total caloric intake (*P* = 0.009) as well as with increased levels of serum triglycerides (*P* < 0.001) (Table 1). Race was also significantly associated with retinol tertiles (*P* < 0.001); being white was associated with higher retinol levels (42% fell in third tertile) while being black was associated with lower levels (56% fell in first tertile). Increasing beta-carotene levels were associated with lower BMI and WHR and higher education. Serum retinol levels were not closely correlated with serum carotenoid levels (*r* ≤ 0.13) nor were serum carotenoids highly correlated with one another (Table 2).

In age- and multivariate-adjusted generalized linear mixed models, taking into account up to 16 repeated measures per subject, serum retinol was positively associated with serum biomarkers of insulin resistance (Table 3). Each *μ*g/mL increase in serum retinol was positively associated with HOMA-IR (*β*:  0.19, *P* = 0.002) and fasting insulin (*β*:  0.2, *P* = 0.0008) and negatively associated with serum SHBG (*β*:  −0.22, *P* < 0.0001). Fasting glucose was not significantly associated with serum retinol (*β*:  −0.014, *P* = 0.74). 

Overall, significant associations between provitamin A carotenoids (*β*-carotene and *β*-cryptoxanthin) and serum biomarkers of insulin resistance were generally not observed, with two exceptions. Although *β*-carotene was not associated with serum insulin (*β*:  −0.014, *P* = 0.49) or HOMA-IR (*β*:  −0.021, *P* = 0.34), it was positively associated with SHBG (*β*:  0.038, *P* = 0.0005). And, while *β*-cryptoxanthin was similarly not associated with HOMA-IR (*β*:  −0.01, *P* = 0.68), it was inversely associated with fasting glucose levels (*β*:  −0.049, *P* = 0.003).

With regards to the other carotenoids, no significant associations in multivariate-adjusted analyses were observed for lutein/zeaxanthin, lycopene, or total carotenoids with any of the biomarkers of insulin resistance. Although lycopene was positively associated with HOMA-IR and insulin in crude models (*P* = 0.02), adjustment for confounders attenuated the associations. Additional adjustment for total cholesterol, alcohol, dietary fiber, and fat intake did not qualitatively affect the results, nor did removal of BMI from the models or substitution of WHR for BMI (results not shown).

## 4. Discussion

 Among healthy premenopausal women not consuming vitamins or supplements in the BioCycle Study, serum retinol was strongly associated with increased HOMA-IR and fasting insulin and decreased SHBG. Although serum carotenoid concentrations were generally not associated with biomarkers of insulin resistance, significant positive associations between *β*-carotene and SHBG and between *β*-cryptoxanthin and fasting plasma glucose were identified.

### 4.1. Serum Retinol

In two National Health and Nutrition Examination Surveys (NHANES) analyses (NHANES III (1988–1994) [15] and NHANES 2001–2006 [7]), no association with hyperglycemia or insulin resistance was found despite associations with the metabolic syndrome. However, differences between the NHANES population and the BioCycle participants should be noted, including the age (20–85 years) of the participants. Moreover, although the NHANES studies controlled for vitamin supplementation in analyses, our participants were not consuming vitamin supplements during study follow-up. This abstinence was reflected in the median levels of serum vitamin A found in BioCycle which were lower in comparison to levels among 20–39-year-old women from the NHANES 2001-2002 survey (38.0 versus 49.1 *μ*g/dL) [33]. 

Serum retinol was not strongly correlated with serum carotenoids in our sample, a finding supportive of nondietary determinants of interindividual variation. Although mechanisms underlying intraindividual homeostatic serum retinol levels are not fully understood, they likely include age, gender, hormones, and genes [3]. However, it* is* known that in individuals of normal vitamin A status, RBP-bound retinol accounts for ~85–90% of vitamin A transported in the blood, and RBP has been validated and used as an indicator of vitamin A status [34, 35]. Thus, the measure of retinol in the BioCycle population of healthy women may have served as a proxy for RBP.

Accordingly, the putative associations between serum retinol and markers of insulin resistance are not wholly unexpected. RBP4, the specific transport protein for retinol [21], is elevated in the serum prior to the development of apparent diabetes and indicative of insulin resistance across subjects exhibiting a range of metabolic profiles [20]. The role of retinol in regulating gene transcription has been proposed as the biological mechanism by which the retinol : RBP : TTR nutrient-protein complex influences insulin and glucose metabolism. Specifically, RPB-bound retinol has been shown to activate plasma membrane protein and vitamin A transporter STRA6, which initiates a signaling pathway that upregulates the transcription of target genes (SCOSs) known to play a critical role in maintaining energy balance and insulin signaling [24].

Previous studies involving healthy participants support associations between RBP4 and insulin resistance. In a small sample (*n* = 67) of healthy women (18–45 years) serum RBP4 concentrations were inversely related to insulin sensitivity assessed by euglycemic hyperinsulinemic clamp [22]. In a larger (*n* = 2614) cross-sectional study, RBP4 levels were positively associated with prediabetes and with several metabolic risk factors, including BMI, waist circumference, hypertension, and plasma lipids [23]. Further, manipulations of GLUT4 expression and RBP4 levels in mice suggest that RBP4 may play a causal role in the pathogenesis of type 2 diabetes [21].

### 4.2. Lycopene

Although adjustment for confounders attenuated statistical significance, positive associations between lycopene and insulin resistance were observed in crude models. Of the major carotenoids, lycopene is the most effective in quenching singlet oxygen [36], and, in our sample, accounted for approximately half of median total serum carotenoid levels. However, based on NHANES (2003–2006) data approximately 85% of lycopene in the American diet is sourced from tomatoes and tomato products (tomatoes, pizza, pasta and pasta mixed dishes, condiments (ketchup), tomato sauces, and soups) [37]. As these foods tend to be high in sodium and fat and low in fiber, dietary patterns associated with lycopene consumption may account for the borderline positive associations observed with insulin resistance.

### 4.3. Provitamin A Carotenoids

Despite the apparent associations between retinol and biomarkers of insulin resistance, similar associations were not observed for the provitamin A (retinol-precursor) carotenoids. In fact, *β*-carotene was not associated with HOMA-IR, insulin, or glucose. While findings from observational studies have produced equivocal results [7, 8], our results agree with the findings from large randomized controlled trials negating a protective role of *β*-carotene in the pathogenesis of insulin resistance [17, 19, 38]. We add to these findings the lack of association also among premenopausal women, a group not well captured in clinical trials.

 On the other hand, the positive association between *β*-carotene and SHBG was significant and persistent. Low SHBG concentrations have been shown to predict the development of type 2 diabetes, independent of glucose and insulin, in premenopausal women and may play a role in its pathogenesis [39, 40]. Other studies of *β*-carotene and SHBG among women are lacking and therefore the association requires replication. In addition, although there is no direct evidence on the possible mechanism connecting beta carotene and SHBG, some indirect observations are worth noting. Evidence from an animal model suggests that *β*-carotene may reduce peroxisome proliferator-activated receptor gamma (PPAR*γ*) activity in adipocytes [41]. If such reduction in PPAR*γ* activity also occurs in the liver, it could explain the concomitant rise in SHBG [42]. Nevertheless, this is a conjecture based on limited evidence and more mechanistic studies are warranted. 

*β*-cryptoxanthin, also a retinol-precursor carotenoid, was inversely associated with circulating glucose levels but was not associated with other biomarkers of insulin resistance. Together with *β*-carotene results, these findings provide weak evidence for a possible protective role of provitamin A carotenoids in insulin resistance in a young, healthy, female cohort. 

The low correlations and differential associations found for retinol and the provitamin A carotenoids may be due to a wide range of factors. The activity and effectiveness of carotenoids as antioxidants depends on numerous factors, including molecular structure, location or site of action within the cell, concentration, reactant properties, partial pressure of oxygen, and interaction with other antioxidants [43]. Furthermore, absorption and conversion to retinol are dependent upon molecular structure and bioavailability as well as individual variation in vitamin A status, body composition, genetics, and physiological state; therefore, conversion efficiency varies considerably, even among relatively homogenous individuals [1, 44, 45]. Thus, it is reasonable and expected that serum carotenoid levels did not correlate strongly with retinol levels. Similarly, the control mechanisms responsible for establishing and maintaining an individual's homeostatic level of circulating retinol have not been fully elucidated and likely involve a combination of factors, including diet composition, gender, and physiologic state [1]. 

### 4.4. Strengths and Limitations

Strengths of this study include the design, which provided multiple measures of retinol and carotenoids, biomarkers of insulin resistance and covariates over time. The multiple measurements decreased the intra-individual and intra-assay variability of both the exposures and the outcomes measured, allowing models to test for small effects. The primary outcome, HOMA-IR, has been validated in assessing insulin resistance based on fasting plasma glucose and serum insulin levels in epidemiological studies [46]. Furthermore, follow-up and compliance rates were high, with 250 women completing visits for 2 menstrual cycles, and 94% of women completing at least 7 visits per cycle. Since women in the BioCycle study refrained from dietary supplement consumption for its duration, this analysis was able to remove possible reverse causality among a subgroup who may have taken supplements due to underlying health conditions. Finally, the completion of up to eight 24-hour dietary recalls provided a reliable measure of confounding dietary factors. 

However, the study was not without limitations. The women who participated were young and healthy, with lower insulin levels than expected in the general population. According to NHANES 1999–2002, mean log fasting insulin levels among nondiabetic women of comparable age range (20–39 years) were (mean ± (SE)) 2.21 (±0.03) mU/L compared to 1.91(±0.01) mU/L in the BioCycle study [47]. Therefore, our study cohort may not be able to describe an association that may be more apparent in a group with higher rates of insulin resistance. Nevertheless, investigating biomarkers of insulin resistance among apparently healthy women of reproductive age has important implications for uncovering factors predictive of and potentially protective against type 2 diabetes.

In light of these considerations, future research is needed to confirm the associations between serum retinol and insulin resistance observed here and further elucidate the relationship between serum antioxidants and type 2 diabetes in women of reproductive age. 

## 5. Conclusion

Our findings suggest that serum carotenoid levels are minimally associated with glucose homeostasis in a young, healthy, female population. However, the notable finding that serum retinol is associated with compromised glucose homeostasis warrants further attention.

## Figures and Tables

**Table 1:** Demographic characteristics of women by tertiles of serum retinol and *β*-carotene.

	Tertiles of serum retinol				Tertiles of serum *β*-carotene			
	T1 (*n* = 84)	T2 (*n* = 85)	T3 (*n* = 84)	*P*	T1 (*n* = 81)	T2 (*n* = 81)	T3 (*n* = 81)	*P*
		*μ*g/mL				*μ*g/mL		
Retinol (*μ*g/mL)	0.30 (0.04)	0.38 (0.02)	0.48 (0.06)	n/a	0.37 (0.09)	0.39 (0.08)	0.39 (0.08)	0.31
*β*-carotene (*μ*g/mL)	0.15 (0.10)	0.22 (0.14)	0.20 (0.14)	0.004	0.08 (0.02)	0.16 (0.02)	0.33 (0.13)	n/a
Age (years)	27.2 (8)	27.2 (8)	27.5 (9)	0.95	26.5 (8.3)	27.2 (8.04)	28.9 (8.4)	0.15
BMI (kg/m^2^)	24.5 (4)	23.3 (3)	24.5 (4)	0.09	24.8 (3.8)	24.4 (4.2)	23.2 (3.6)	0.02
WHR (cm)	0.76 (0.05)	0.74 (0.04)	0.76 (0.05)	0.002	0.76 (0.07)	0.75 (0.04)	0.74 (0.05)	0.02
Race, *n* (%)								
White	38 (45%)	50 (59%)	63 (75%)		49 (60%)	53 (65%)	46 (57%)	
Black	36 (43%)	17 (20%)	11 (13%)	<0.001	20 (25%)	13 (16%)	13 (16%)	0.21
Other	10 (12%)	18 (21%)	10 (12%)		12 (15%)	9 (19%)	22 (27%)	
Physical activity, *n* (%)								
Low	12 (14%)	6 (7%)	7 (8%)		9 (11%)	8 (10%)	6 (7%)	
Moderate	28 (33%)	36 (42%)	26 (31%)	0.27	32 (40%)	24 (30%)	32 (40%)	0.55
High	44 (52%)	43 (51%)	51 (61%)		40 (49%)	49 (60%)	43 (53%)	
Education, *n* (%)								
≤ HS	11 (13%)	8 (8%)	13 (15%)	0.53	15 (17%)	11 (14%)	6 (7%)	0.002
Some college	38 (45%)	30 (35%)	32 (38%)		42 (52)	30 (37%)	21 (26%)	
College degree	27 (32%)	38 (45%)	28 (33%)		19 (23)	32 (40%)	40 (49%)	
Graduate school	8 (10%)	9 (11%)	11 (13%)		6 (7%)	8 (10%)	14 (17%)	
Smoking, *n* (%)								
Nonsmoker	31 (37%)	37 (44%)	29 (35%)		26 (32%)	30 (37%)	38 (50%)	
Passively exposed	48 (57%)	47 (55%)	51 (61%)	0.41^2^	50 (62%)	47 (58%)	42 (52%)	0.22^2^
Smoker	5 (6%)	1 (1%)	4 (5%)		5 (6%)	4 (5%)	1 (1%)	
Total calories (kcal)	1510 (314)	1614 (352)	1676 (348)	0.009	1585 (317)	1613 (382)	1636 (361)	0.65
Total fat (% kcal)	34.0 (6)	33.4 (5)	34.1 (6)	0.71	34 (5)	34 (6)	33 (5)	0.34
Triglycerides (mg/dL)^1^	47 (38–66)	51 (38–70)	64 (47–88)	<0.001	60 (44–81)	52 (39–67)	51 (38–73)	0.21
Total cholesterol (mg/dL)^1^	165 (147–189)	165 (148–182)	174 (152–193)	0.19	164 (148–179)	165 (151–189)	172 (150–193)	0.11
HDL (mg/dL)^1^	49 (41–58)	51 (44–60)	53 (43–62)	0.22	47 (39–58)	50 (44–59)	51 (45–61)	0.05
LDL (mg/dL)^1^	102 (88–124)	100 (86–117)	104 (89–125)	0.68	102 (88–115)	104 (86–127)	102 (89–124)	0.18

Mean (SD) unless indicated.

Abbreviations. WHR: waist-to-hip ratio; HS: high school.

^1^Median (25th–75th percentile).

^2^Exact Test.

**Table 2:** Spearman correlation coefficients between serum carotenoids and retinol^1^.

	*β*-cryptoxanthin	Lutein/zeaxanthin	Lycopene	Retinol
*β*-carotene	0.57	0.47	0.26	0.08
*β*-cryptoxanthin	—	0.44	0.15	0.13
Lutein/zeaxanthin	—	—	0.12	0.13
Lycopene	—	—	—	0.11

^1^Adjusted for age, race, and triglycerides.

**Table 3:** Mean log levels (95% CI) of biomarkers of insulin resistance per unit increase in level of serum carotenoids and retinol (*μ*g/mL)^1^.

	Model 1^2^	*P* value	Model 2^3^	*P* value
HOMA				
*β*-carotene	−0.016 (−0.061, 0.029)	0.48	−0.021 (−0.064, 0.022)	0.34
*β*-cryptoxanthin	−0.024 (−0.074, 0.026)	0.35	−0.010 (−0.058, 0.038)	0.68
Lutein/zeaxanthin	−0.007 (−0.055, 0.042)	0.79	−0.011 (−0.058, 0.036)	0.64
Lycopene	0.054 (0.009, 0.098)	0.02	0.039 (−0.004, 0.083)	0.07
Total carotenoids	0.078 (−0.004, 0.16)	0.06	0.056 (−0.023, 0.134)	0.17
Retinol	0.35 (0.23, 0.48)	<0.0001	0.19 (0.07, 0.32)	0.002
Glucose (mmol/L)				
*β*-carotene	−0.021 (−0.050, 0.008)	0.15	−0.024 (−0.053, 0.005)	0.11
*β*-cryptoxanthin	−0.048 (−0.080, −0.016)	0.003	−0.049 (−0.081, −0.016)	0.003
Lutein/zeaxanthin	−0.011 (−0.043, 0.021)	0.50	−0.012 (−0.045, 0.020)	0.45
Lycopene	0.018 (−0.012, 0.048)	0.24	0.018 (−0.012, 0.048)	0.23
Total carotenoids	−0.009 (−0.063, 0.044)	0.73	−0.011 (−0.064, 0.043)	0.70
Retinol	0.021 (−0.061, 0.102)	0.62	−0.014 (−0.098, 0.070)	0.74
Insulin (pmol/L)				
*β*-carotene	−0.010 (−0.052, 0.033)	0.65	−0.014 (−0.055, 0.026)	0.49
*β*-cryptoxanthin	−0.012 (−0.059, 0.035)	0.61	0.001 (−0.044, 0.047)	0.95
Lutein/zeaxanthin	−0.005 (−0.050, 0.041)	0.85	−0.008 (−0.052, 0.036)	0.72
Lycopene	0.049 (0.007, 0.091)	0.02	0.036 (−0.005, 0.077)	0.08
Total carotenoids	0.079 (0.002, 0.156)	0.05	0.059 (−0.015, 0.133)	0.12
Retinol	0.353 (0.234, 0.472)	<0.0001	0.20 (0.08, 0.31)	0.0008
SHBG (nmol/L)				
*β*-carotene	0.039 (0.018, 0.060)	0.0004	0.038 (0.017, 0.060)	0.0005
*β*-cryptoxanthin	0.007 (−0.017, 0.032)	0.54	0.007 (−0.017, 0.031)	0.58
Lutein/zeaxanthin	0.002 (0.021, 0.024)	0.87	0.001 (−0.022, 0.023)	0.94
Lycopene	−0.017 (−0.037, 0.004)	0.11	−0.018 (−0.038, 0.003)	0.09
Total carotenoids	−0.004 (−0.043, 0.035)	0.86	−0.006 (−0.045, 0.033)	0.76
Retinol	−0.214 (−0.274, −0.154)	<0.0001	−0.22 (−0.28, −0.16)	<0.0001

^1^All variables are log-transformed, with the exception of glucose.

^2^Model 1 adjusts for age.

^3^Model 2 adjusts for age, race, smoking, physical activity, BMI, triglycerides, and total average energy intake.
